# Split nitrogen application increases grain yield of densely planted summer maize by delaying post-silking leaf senescence

**DOI:** 10.3389/fpls.2026.1871425

**Published:** 2026-08-04

**Authors:** Zhen Guo, Guangzhou Liu, Diangang Guo, Deming Liu, Zhihua Wu, Zehua Ma, Zhen Gao, Dahong Bian, Yanhong Cui, Xiong Du

**Affiliations:** State Key Laboratory of North China Crop Improvement and Regulation, Key Laboratory of Crop Growth Regulation of Hebei Province, College of Agronomy, Hebei Agricultural University, Baoding, China

**Keywords:** dense planting, drip fertigation, leaf senescence, split nitrogen application, summer maize, yield formation

## Abstract

Increasing planting density can raise summer maize yield, but it also intensifies competition for light, water, and nutrients within the canopy, accelerating post-silking leaf senescence and reducing assimilatory capacity. Drip fertigation enables precise water and nitrogen supply, but the optimal nitrogen application frequency for summer maize remains unclear in the North China Plain. Therefore, a two-year field experiment was conducted in 2024 and 2025 using two planting densities (75,000 and 97,500 plants ha^-^¹), a conventional single nitrogen application control, and several split nitrogen treatments under the same total nitrogen rate. Across the two-year average, split nitrogen application increased grain yield and 1000-kernel weight, and the four-split treatment produced the highest grain yield (14.4t ha^-^¹). At 75,000 and 97,500 plants ha^-^¹, the two-year average grain yield under the four-split treatment increased by 11.8% and 13.0%, respectively, and the 1000-kernel weight increased by 6.0% and 10.0%, respectively, compared with the corresponding control. Based on the two-year average, the four-split treatment also delayed leaf senescence onset by 2.1 and 6.0d, delayed the time to maximum senescence rate by 11.5 and 12.5d, and reduced the mean senescence rate by 18.0% and 20.8%, respectively. Overall, split nitrogen application, especially the four-split regime, delayed post-silking leaf senescence, improved late-season physiology, and increased grain yield in densely planted summer maize.

## 1 Introduction

Maize is one of the world’s major crops for food, feed, and industrial use (Erenstein et al., 2022). As constraints on arable land resources continue to intensify and the demand for higher yield per unit area keeps increasing, improving yield potential per unit area has become an important goal in maize production (Wang et al., 2023b; Du et al., 2024b). Increasing planting density can enhance canopy light interception and land use efficiency, and is therefore an important agronomic practice for increasing maize yield potential (Shao et al., 2024a; Wu et al., 2024; Zhang et al., 2024). However, excessive canopy crowding also intensifies competition for light, water, and nutrients, which may shorten the functional duration of leaves after silking and weaken assimilate supply during grain filling (Shah et al., 2021; Ding et al., 2025; Hong et al., 2025). Therefore, post-silking leaf senescence represents a key physiological bottleneck limiting yield improvement under dense planting conditions (Li et al., 2022b).

Post-silking leaf senescence is not only a decline in green leaf area, but also a coordinated physiological process involving chlorophyll degradation, oxidative stress, protein turnover, and nitrogen remobilization from vegetative organs to developing grains (Lan et al., 2024; Cheng et al., 2025; Wei et al., 2025). During senescence, excessive accumulation of reactive oxygen species can impair membrane stability and accelerate chloroplast degradation, while antioxidant enzymes such as superoxide dismutase, peroxidase, and catalase help scavenge reactive oxygen species and alleviate oxidative damage (Li et al., 2022a). Malondialdehyde, as a product of membrane lipid peroxidation, is commonly used to indicate the degree of cellular membrane damage during leaf senescence. Meanwhile, soluble protein content and leaf nitrogen status are closely associated with the maintenance of photosynthetic enzymes, chlorophyll-protein complexes, and stay-green capacity (Qu et al., 2023). In particular, the onset time of leaf senescence (Ts) indicates when the post-silking green leaf area begins to decline rapidly and therefore reflects the duration of functional canopy maintenance during grain filling. Therefore, indicators such as post-silking leaf area duration, chlorophyll content, antioxidant enzyme activities, MDA content, soluble protein content, and nitrogen accumulation can jointly reflect the physiological progression of leaf senescence and its contribution to grain filling (Jiang et al., 2025).

Nitrogen management is an important agronomic approach for regulating these physiological processes in densely planted maize. Adequate nitrogen supply during the reproductive stage can maintain leaf nitrogen concentration, delay chlorophyll degradation, sustain photosynthetic enzyme activity, and reduce premature remobilization of nitrogen from leaves to grains (Liu et al., 2023; Guo et al., 2025). In contrast, insufficient late-season nitrogen supply may accelerate leaf nitrogen export, weaken antioxidant defense, increase membrane lipid peroxidation, and consequently promote premature senescence (Liao et al., 2024; Du et al., 2024b; Cao et al., 2026). Therefore, optimizing nitrogen application timing under drip fertigation may delay senescence by coordinating nitrogen uptake, nitrogen remobilization, antioxidant protection, and source–sink balance during grain filling (Zheng et al., 2023). Previous studies have shown that drip fertigation can couple irrigation and fertilization, achieve coordinated water and nitrogen supply in the root zone through precise regulation of water and nitrogen inputs, and improve water and nitrogen use efficiency in maize (Ning et al., 2024). Optimized nitrogen management under drip fertigation also helps improve post-silking nitrogen uptake, maintain canopy photosynthetic function, promote dry matter accumulation, and increase yield potential (Guo et al., 2022; Cheng et al., 2023; Lai et al., 2025). Nitrogen application timing is likewise an important factor affecting nitrogen use efficiency and yield formation in maize. Previous studies have shown that a moderate delay in nitrogen application is beneficial for improving nitrogen supply during the reproductive stage, maintaining post-silking leaf function, and promoting grain filling and yield formation (Mueller et al., 2017; Maltese et al., 2024; Yin et al., 2024; Zhao et al., 2026). Under drip fertigation, the advantage of this stage-specific and demand-driven nitrogen supply strategy becomes more evident. However, the optimal nitrogen application frequency and timing are not fully consistent across different ecological regions and cropping systems. For example, Mao et al. (2022) found in an irrigated spring maize system in Xinjiang that increasing the number of topdress nitrogen applications helped maintain higher photosynthetic potential after silking and promoted dry matter accumulation, with the eight-application treatment showing the best performance. However, this result cannot be directly extrapolated to summer maize production on the North China Plain. Compared with spring maize in Xinjiang, summer maize in the North China Plain is grown in a wheat–maize double-cropping system with a shorter growing season and greater overlap between the reproductive stage and the hot, rainy summer period. Under high planting density, the canopy is more crowded, and competition for light and nitrogen during the grain-filling stage may be intensified. Therefore, the optimal nitrogen-splitting frequency may differ between these two production systems, and it is necessary to determine a region-specific split nitrogen regime for densely planted summer maize under drip fertigation (Mao et al., 2022).

Accordingly, it is necessary to clarify the effects of different split nitrogen application regimes on post-silking leaf senescence, post-silking dry matter production, and yield formation under dense planting of summer maize, which is of great significance for improving maize yield in this region. In this study, two planting densities and four nitrogen application regimes were compared under drip fertigation. Unlike previous studies that mainly focused on yield or nitrogen-use efficiency, this study integrates Logistic-derived senescence parameters with ear-leaf physiological traits and organ-level nitrogen accumulation to explain how split nitrogen application regulates post-silking source maintenance under dense planting. The objectives of this study were to: 1) determine the effects of nitrogen application frequency on grain yield and yield components under two planting densities; 2) quantify post-silking leaf senescence dynamics using Logistic-derived parameters; and 3) clarify whether split nitrogen application delays leaf senescence by maintaining leaf photosynthetic capacity, enhancing antioxidant defense, reducing membrane lipid peroxidation, and improving nitrogen accumulation and remobilization during grain filling.

## 2 Materials and methods

### 2.1 Site description

**Figure 1:**
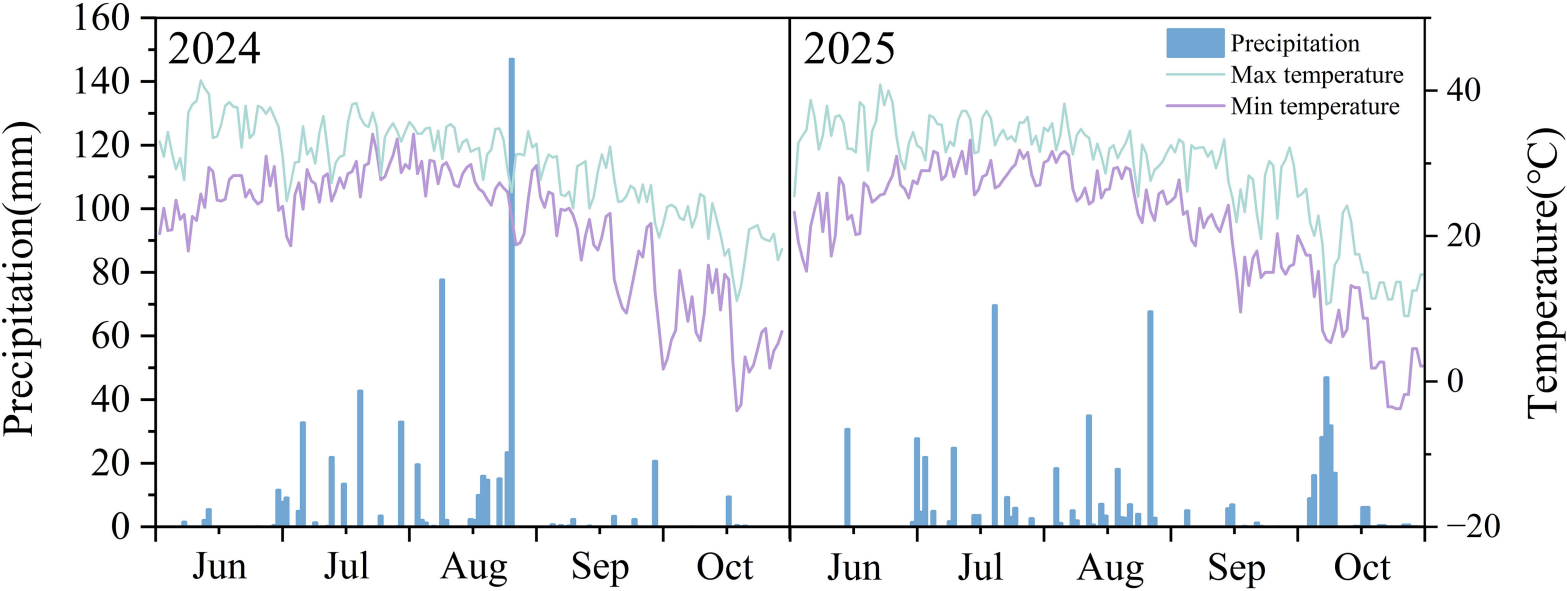
Daily precipitation and maximum and minimum air temperatures during the maize growing seasons in 2024 and 2025.

The experiment was conducted in 2024 and 2025 at the Qingyuan Experimental Station of Hebei Agricultural University in Baoding, Hebei, China (38°49′N, 115°26′E). The soil was loamy fluvo-aquic soil, with 14.45g kg^-^¹ organic matter, 69.63 mg kg^-^¹ alkali-hydrolyzable nitrogen, 29.34 mg kg^-^¹ available phosphorus, and 126.85 mg kg^-^¹ available potassium. The mean air temperature and daily precipitation during the summer maize growing seasons in the two years are shown in **Figure 1**. The mean temperatures during the maize growing period were 25.8°C in 2024 and 25.1°C in 2025, and the corresponding precipitation amounts were 545.6mm and 570.0mm, respectively.

### 2.2 Experiment design

The hybrid MY73 was used in this experiment. Two planting densities were established: 75,000 plants ha^-^¹ (S) and 97,500 plants ha^-^¹ (D). Under the same total nitrogen rate of 270kg ha^-^¹, a conventional single basal nitrogen application treatment was used as the control (CK), together with four split nitrogen application treatments. The specific nitrogen application stages and rates are shown in **Table 1**.

**Table 1:** Experimental design of plant density and split nitrogen application treatments.

Density (plants ha^-1^)	Treatment	Basal N rate (kg ha^-1^)	Topdressing N rate(kg ha^-1^)				
			V12-V14	R1	10–14 DAS	18–22 DAS	
75,000	CK1	270					
	SN1	54	216				
	SN2	54	108	108			
	SN3	54	72	72	72		
	SN4	54	54	54	54		54
97,500	CK2	270					
	DN1	54	216				
	DN2	54	108	108			
	DN3	54	72	72	72		
	DN4	54	54	54	54		54

CK1 and SN1–SN4 denote treatments under S, whereas CK2 and DN1–DN4 denote treatments under D. CK, conventional single basal N application; N1–N4, split N application at different growth stages. V12–V14, large bell stage; R1, silking; DAS, days after silking.

The experiment was arranged in a randomized block design with three replicates, comprising a total of 30 plots. Each plot measured 10m × 15m. The row spacing configuration was 40cm + 80cm, and drip irrigation belts were laid in the narrow rows. One fertilizer tank was installed for each plot to ensure accurate nitrogen application at different stages. Except for differences in the timing of nitrogen application, all treatments received the same total nitrogen rate, and topdressed nitrogen was applied with irrigation. The control treatment was irrigated simultaneously at the corresponding stages to minimize the interference of irrigation differences with treatment effects. A total of 60mm of irrigation water was applied during the whole growing season through drip irrigation in five events of 12mm each, including one emergence irrigation and four subsequent irrigation/fertigation events at V12–V14, R1, 10–14 days after silking, and 18–22 days after silking. Topdressed nitrogen was applied with irrigation according to the treatment schedule shown in **Table 1**. All treatments, including CK, received the same irrigation schedule, and irrigation water alone was applied when no nitrogen topdressing was scheduled, thereby avoiding confounding nitrogen timing with water supply. All treatments received 135kg ha^-^¹ P_2_O_5_ and 150kg ha^-^¹ K_2_O as basal fertilizer, and urea (46% N) was used for topdressed nitrogen. Sowing was carried out on June 19, 2024 and June 18, 2025, and harvest was conducted on October 11, 2024 and October 18, 2025. Field management practices for pests, diseases, weeds, and other agronomic measures were consistent with local high-yield cultivation conditions.

### 2.3 Measurements and calculations

#### 2.3.1 Post-silking photosynthetic potential, leaf senescence characteristics, and parameter calculation

Leaf area (LA, cm² plant^-^¹) was calculated as maximum leaf length × maximum leaf width × a coefficient, where the coefficient was 0.75 for fully expanded leaves and 0.50 for incompletely expanded leaves. Leaf area index (LAI) was calculated as the total leaf area per unit land area. Post-silking photosynthetic potential (LAD) was calculated using Equation 1 as follows:

(1) $$LAD=\left(\frac{LAI_{1}+LAI_{2}}{2}\right)\times \left(t_{2}-t_{1}\right)$$

where LAI_1_ and t_1_ represent the leaf area index and time at silking, respectively, and LAI_2_ and t_2_ represent the leaf area index and time at maturity, respectively.

According to Xu et al. (2024), leaf area was measured at silking and at 10-d intervals thereafter until physiological maturity. Three tagged plants were continuously sampled in each plot. The green leaf area of each plant was measured, and relative green leaf area (RGLA) was calculated as the percentage of green leaf area at each sampling date relative to that at silking, with the value at silking set to 100%. A Logistic equation was used to fit the dynamic changes in relative green leaf area (RGLA) after silking, and the goodness of fit was evaluated by the coefficient of determination (R²). RGLA at silking was set to 100%, and that at complete yellowing was set to 0. Based on the fitted parameters, the onset time of leaf senescence (Ts), mean senescence rate (V), maximum senescence rate (Vmax), and the time at which the maximum senescence rate occurred (Tmax) were calculated. Ts was defined as the time when RGLA declined to 95%. Throughout the manuscript, Ts, V, Vmax, and Tmax were used to denote the characteristic parameters of leaf senescence. The temporal dynamics of post-silking RGLA were fitted using Equation 2, and V, Vmax, and Tmax were calculated using Equations 3–5:

(2) $$y=\frac{a}{1+e^{cx-b}}$$

(3) $$V=\frac{RGLA_{s}-RGLA_{m}}{t}$$

(4) $$V_{max}=\frac{ac}{4}$$

(5) $$T_{max}=\frac{b}{c}$$

where y is the predicted relative green leaf area (%), x is days after silking, a is the upper asymptote of RGLA and was fixed at 100 in this study, b is a location parameter related to the timing of senescence, and c is the senescence rate coefficient. RGLAs is the relative green leaf area (%) at silking, RGLAm is the relative green leaf area (%) at maturity, and t is the time interval (d) from silking to maturity. The onset time of leaf senescence (Ts) was defined as the time corresponding to x when y = 95%.

#### 2.3.2 Determination of photosynthetic pigment contents, antioxidant enzyme activities, and soluble protein content

At the silking and maturity stages, three plants with uniform growth were selected from each plot, and the ear leaves were collected. After removal of the midrib, the samples were wrapped in aluminum foil and immediately frozen in liquid nitrogen for the determination of photosynthetic pigment contents, antioxidant enzyme activities, and soluble protein content in the ear leaves. Photosynthetic pigment contents were determined using the 95% ethanol extraction method. Briefly, 0.5g of leaf tissue was immersed in 10 mL of 95% ethanol and extracted in the dark for 36h until the leaves were completely decolorized and turned white. The absorbance of the extract was then measured at 665, 649, and 470 nm, and the contents of chlorophyll a (Chla), chlorophyll b (Chlb), carotenoids (Car), and total chlorophyll (Chl) were calculated (Xing et al., 2024). The pigment concentrations were expressed on a fresh-weight basis as mg g^-^¹ FW. The pigment concentrations were calculated using Equations 6–9:

(6) $$Chl_{a}=13.95\times A_{665}-6.88\times A_{649}$$

(7) $$Chl_{b}=24.96\times A_{649}-7.32\times A_{665}$$

(8) $$Car=\frac{1000\times A_{470}-2.05\times Chl_{a}-114.8\times Chl_{b}}{245}$$

(9) $$Chl=Chl_{a}+Chl_{b}$$

Superoxide dismutase (SOD) activity was determined by the nitro blue tetrazolium (NBT) photoreduction method, peroxidase (POD) activity was determined by the guaiacol colorimetric method, catalase (CAT) activity was determined by ultraviolet spectrophotometry, malondialdehyde (MDA) content was determined by the thiobarbituric acid colorimetric method, and soluble protein content was determined by the Coomassie brilliant blue method.

#### 2.3.3 Population dry matter accumulation and nitrogen accumulation

At the silking stage (R1) and maturity stage (R6), three plants with uniform growth were selected from each plot and separated into three parts: stem, leaf, and ear. The samples were oven-dried at 105°C for 30min to deactivate enzymes, and then dried at 80°C to constant weight. The dry matter of each organ was weighed and recorded. After dry weight determination, the dried samples were ground and sieved to prepare samples for total nitrogen analysis. Total nitrogen content in maize plants was determined by the H_2_SO_4_–H_2_O_2_ digestion-colorimetric method. Briefly, 0.200g of dried and ground sample was accurately weighed into a digestion tube, with three replicates for each sample. Then, 1 mL of distilled water and 8 mL of concentrated sulfuric acid were added for predigestion, followed by the addition of an appropriate amount of H_2_O_2_ for further digestion until the digest became clear and transparent. After volume adjustment, the digest was analyzed colorimetrically using an automated chemical analyzer to calculate total nitrogen content in the plant.

#### 2.3.4 Grain yield

At maturity, ears were harvested from the central three rows over a 5-m length in each plot, excluding border plants. Based on the mean row spacing of 0.60m under the wide–narrow row configuration, the harvest area was 9.0 m². After the number of effective ears was counted, 10 ears were randomly sampled from the harvested ears of each plot for yield-component analysis. The number of rows per ear and kernels per row were determined. After measurement, the sampled ears were hand-shelled, and the grains were dried at 80°C to constant weight to determine grain moisture content. Grain yield was finally adjusted to a standard moisture content of 14%.

### 2.4 Statistical analysis

The original data were organized using Excel 2021. A three-way ANOVA was performed with year, planting density, and nitrogen regime as fixed effects. Replicate blocks within each year were treated as random effects. Means were compared using the LSD test at P< 0.05. For differences among nitrogen treatments within the same year and planting density, multiple comparisons were conducted using the LSD test after the analysis of variance reached a significant level, with the significance level set at P< 0.05. Statistical analyses were performed using SPSS 26.0. Data related to leaf senescence were fitted with Logistic curves using R 4.4.2, and the parameters Ts, V, Vmax, and Tmax were calculated based on the fitting results. Figures were generated using R 4.4.2 and Origin 2024.

## 3 Results

### 3.1 Grain yield and its components

Analysis of variance showed that year and planting density had extremely significant effects on grain yield (P< 0.001), whereas nitrogen treatment and the year × nitrogen treatment interaction had significant effects (P< 0.05) (**Table 2**). Year, planting density, nitrogen treatment, and the planting density × nitrogen treatment interaction had extremely significant effects on 1000-kernel weight, while the year × planting density × nitrogen treatment interaction had a significant effect. Kernels per ear were extremely significantly affected (P < 0.001) only by planting density, whereas ears per unit area were extremely significantly affected by planting density, nitrogen treatment, and the year × planting density interaction.

**Table 2:** Effects of plant density and split nitrogen application on grain yield and yield components in 2024 and 2025.

Year	Density (plants ha^-1^)	Treatment	Ear number	Kernel number	1000-kernel weight (g)	Yield (t ha^-1^)
2024	75,000	CK1	7.65 ± 0.41 a	539 ± 36 a	280.2 ± 6.9 c	11.3 ± 0.1 c
		SN1	7.66 ± 0.16 a	534 ± 14 a	283.6 ± 2.0 b	11.6 ± 0.3 c
		SN2	7.75 ± 0.16 a	544 ± 13 a	288.7 ± 7.2 ab	12.1 ± 0.1 bc
		SN3	7.63 ± 0.17 a	543 ± 23 a	292.1 ± 7.3 a	12.4 ± 0.2 ab
		SN4	7.69 ± 0.16 a	560 ± 10 a	302.3 ± 3.5 a	12.9 ± 0.6 a
	97,500	CK2	9.52 ± 0.41 a	470 ± 14 b	245.3 ± 6.9 b	11.7 ± 0.2 d
		DN1	9.43 ± 0.16 a	493 ± 20 ab	249.3 ± 3.1 ab	11.8 ± 0.2 cd
		DN2	9.54 ± 0.70 a	509 ± 22 a	248.3 ± 2.4 ab	12.2 ± 0.3 c
		DN3	9.54 ± 0.16 a	519 ± 33 a	250.9 ± 3.2 ab	12.7 ± 0.2 b
		DN4	9.54 ± 0.16 a	518 ± 40 a	275.5 ± 3.5 a	13.3 ± 0.2 a
2025	75,000	CK1	7.28 ± 0.17 a	513 ± 20 a	334.9 ± 3.5 c	12.5 ± 0.1 d
		SN1	7.45 ± 0.47 a	517 ± 90 a	330.3 ± 5.2 c	12.7 ± 0.2 c
		SN2	7.45 ± 0.26 a	506 ± 12 ab	344.6 ± 7.5 b	13.0 ± 0.3 bc
		SN3	7.45 ± 0.26 a	493 ± 20 b	358.7 ± 7.0 a	13.2 ± 0.3 b
		SN4	7.45 ± 0.26 a	520 ± 50 a	351.6 ± 3.5 ab	13.7 ± 0.1 a
	97,500	CK2	9.48 ± 0.20 a	459 ± 90 ab	316.2 ± 4.4 c	13.8 ± 0.2 e
		DN1	9.49 ± 0.74 a	458 ± 20 b	330.7 ± 9.6 b	14.0 ± 0.4 cd
		DN2	9.67 ± 0.11 a	448 ± 13 b	330.4 ± 7.8 b	14.2 ± 0.3 bc
		DN3	9.67 ± 0.11 a	445 ± 10 b	335.3 ± 4.5 b	14.5 ± 0.2 b
		DN4	9.66 ± 0.88 a	449 ± 50 a	347.4 ± 2.4 a	15.5 ± 0.1 a
	Y		NS	NS	***	***
	D		***	***	***	***
	N		***	NS	***	*
	Y×D		***	NS	NS	NS
	Y×N		NS	NS	NS	*
	D×N		NS	NS	***	NS
	Y×D×N		NS	NS	*	NS

Different lowercase letters indicate significant differences among treatments within the same year and planting density according to the LSD test at P< 0.05. *, **, and *** indicate significance at P< 0.05, P< 0.01, and P< 0.001, respectively; ns indicates no significant difference (P > 0.05). S, 75,000 plants ha^-^¹; D, 97,500 plants ha^-^¹; Y, year; D, planting density; N, number of nitrogen applications.

On average across the two years, grain yield at 97,500 plants ha^-^¹ was 6.6% higher than that at 75,000 plants ha^-^¹, whereas 1000-kernel weight and kernels per ear were reduced by 7.5% and 9.5%, respectively. Under both planting densities, the four-split treatment produced the highest grain yield. Compared with the corresponding control, SN4 and DN4 increased grain yield by 11.8% and 13.0%, respectively. The absolute yield increment under DN4 was numerically greater than that under SN4, although the density × nitrogen interaction for grain yield was not significant. In contrast, split nitrogen application had relatively small effects on ears per unit area and kernels per ear.

### 3.2 Dry matter accumulation

**Figure 2:**
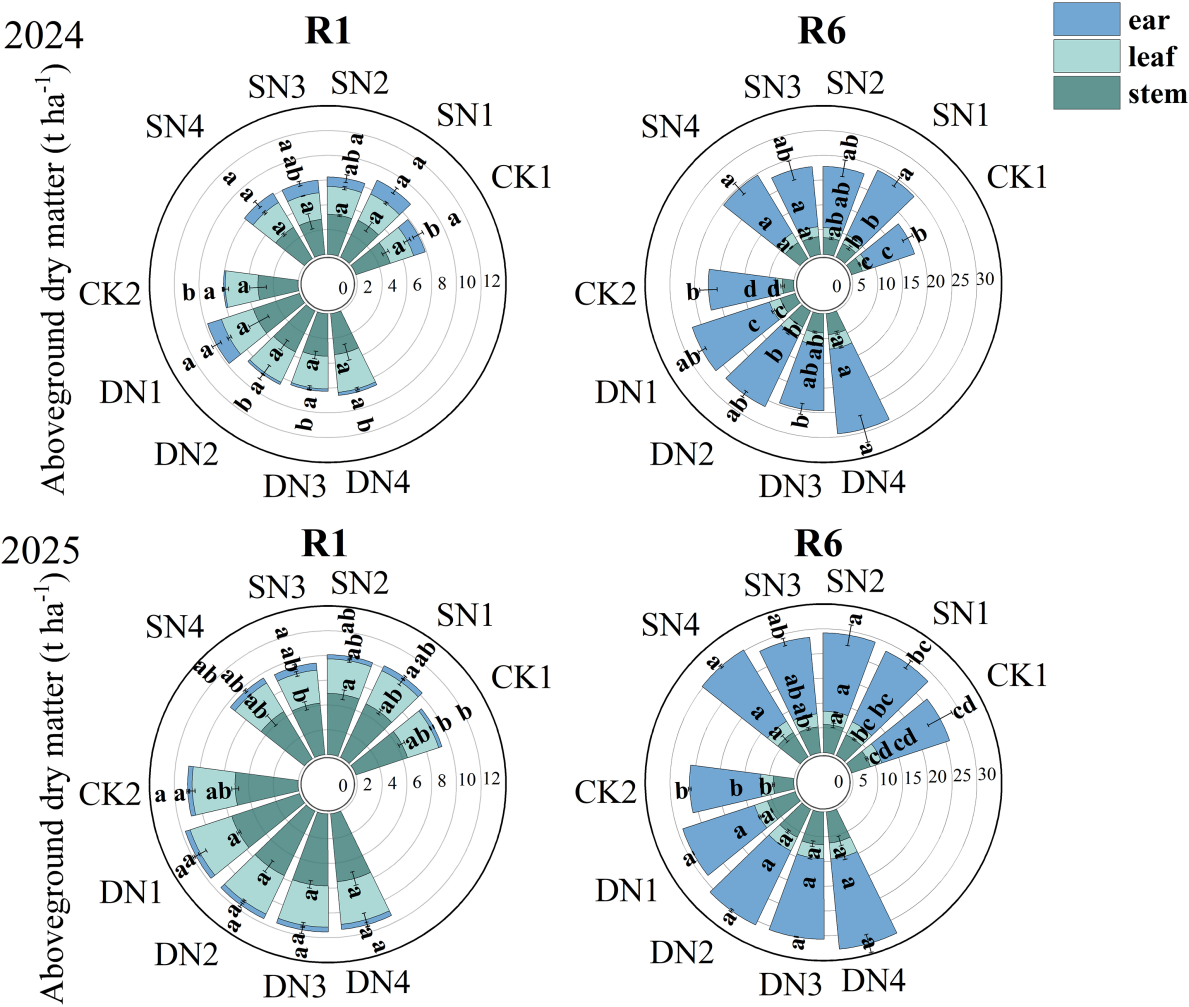
Aboveground dry matter accumulation of stem, leaf, and ear at silking (R1) and physiological maturity (R6) in 2024 and 2025 under different nitrogen application regimes and plant densities. S, 75,000 plants ha^-^¹; D, 97,500 plants ha^-^¹. CK1 and SN1–SN4 denote treatments under S, whereas CK2 and DN1–DN4 denote treatments under D. CK, conventional single basal N application; N1–N4, split N application at different growth stages. Values are means ± SE (n = 3). Different lowercase letters indicate significant differences among treatments within the same year, density, and growth stage at P< 0.05.

Split nitrogen application generally increased aboveground dry matter accumulation and the effect was more evident at maturity than at R1 (**Figure 2**). At silking, treatment differences were relatively small and varied between years and planting densities. At maturity, the advantage of split nitrogen application was mainly reflected in greater ear dry matter accumulation. In 2024, SN1 and SN4 increased ear dry matter accumulation by 32.3% and 28.4%, respectively, compared with CK1, while DN4 produced the highest ear dry matter accumulation under the high-density condition. In 2025, the four-split treatment showed the greatest ear dry matter accumulation under both planting densities, increasing by 14.3% and 21.8% over the corresponding controls under the S and D densities, respectively.

### 3.3 Post-silking photosynthetic potential

**Figure 3:**
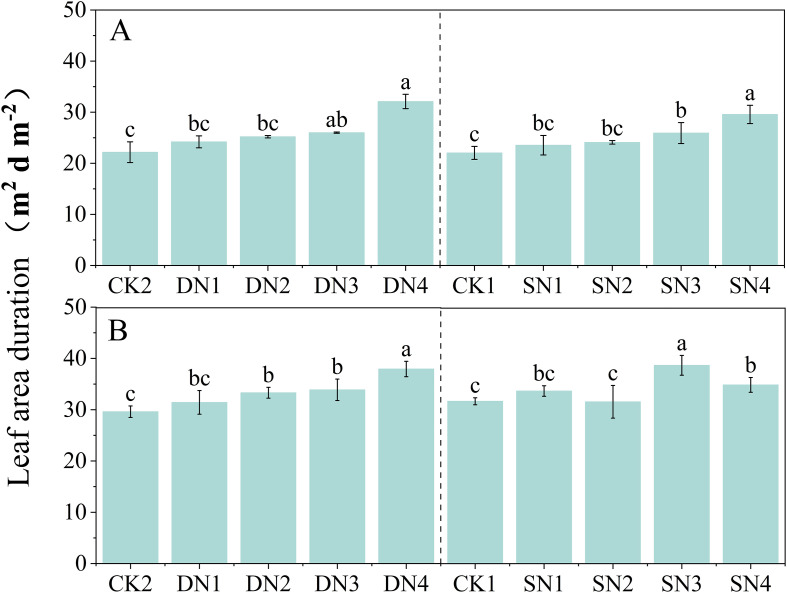
Leaf area duration from silking to physiological maturity in 2024 and 2025 under different nitrogen application regimes and plant densities. S, 75,000 plants ha^-^¹; D, 97,500 plants ha^-^¹; **(A)**, 2024; **(B)**, 2025. CK1 and SN1–SN4 denote treatments under S, whereas CK2 and DN1–DN4 denote treatments under D. CK, conventional single basal N application; N1–N4, split N application at different growth stages. Values are means ± SE (n = 3). Different lowercase letters indicate significant differences among treatments within the same year and density at P< 0.05.

Post-silking photosynthetic potential was generally higher in 2025 than in 2024 and increased with nitrogen application frequency under both planting densities (**Figure 3**). The four-split treatment maintained the highest or a relatively high photosynthetic potential across years. Compared with the corresponding controls, SN4 and DN4 increased post-silking photosynthetic potential by 25.5% and 30.9% in 2024, respectively, while DN4 increased it by 21.6% in 2025.

### 3.4 Photosynthetic pigment contents in the ear leaf

**Figure 4:**
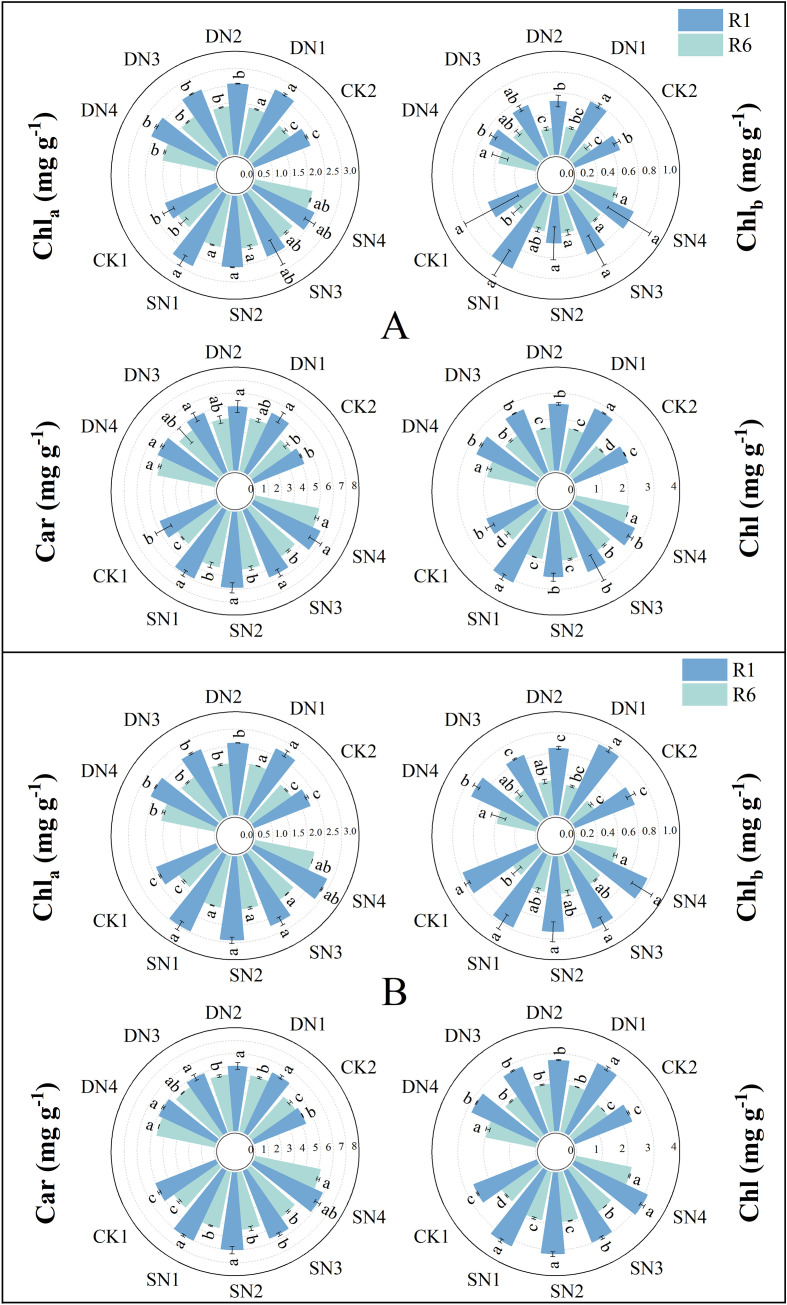
Chlorophyll a, chlorophyll b, carotenoid, and total chlorophyll contents in ear leaves at silking (R1) and physiological maturity (R6) in 2024 and 2025 under different nitrogen application regimes and plant densities. S, 75,000 plants ha^-^¹; D, 97,500 plants ha^-^¹. **(A)**, 2024; **(B)**, 2025. CK1 and SN1–SN4 denote treatments under S, whereas CK2 and DN1–DN4 denote treatments under D. CK, conventional single basal N application; N1–N4, split N application at different growth stages. Values are means ± SE (n = 3). Different lowercase letters indicate significant differences among treatments within the same year, density, and sampling stage at P< 0.05.

Photosynthetic pigment contents in the ear leaf declined from silking to maturity across all treatments (**Figure 4**). Compared with the silking stage, the response of pigment contents to nitrogen application frequency was more evident at maturity. At maturity, split nitrogen application, especially the four-split treatment, maintained higher chlorophyll and carotenoid contents under both planting densities. In 2024, DN4 increased total chlorophyll and carotenoid contents by 19.0% and 18.2%, respectively, compared with CK2. Similar trends were observed in 2025, indicating that split nitrogen application helped maintain photosynthetic pigment levels during late growth.

### 3.5 Nitrogen accumulation

**Figure 5:**
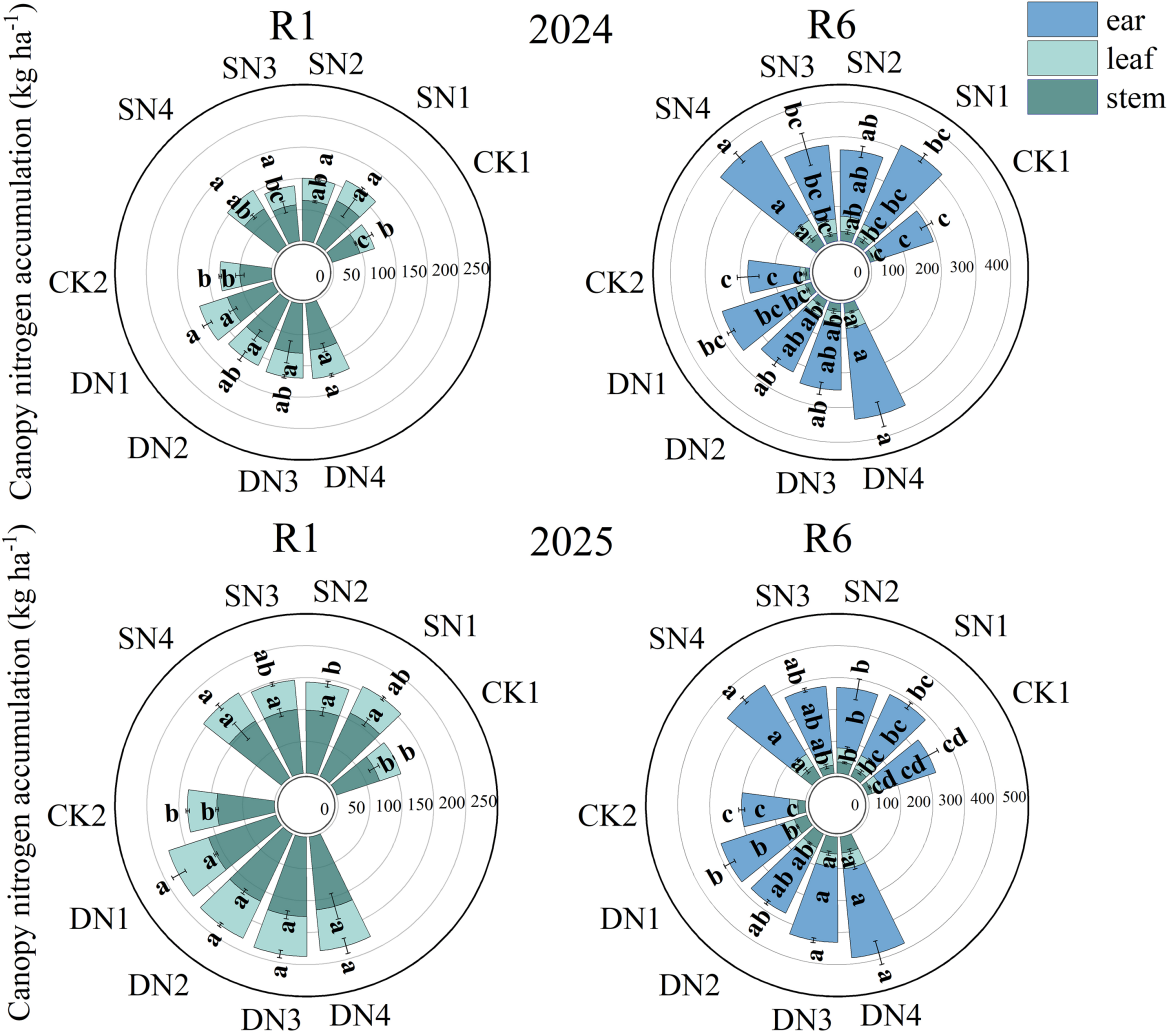
Population nitrogen accumulation in stem, leaf, and ear at silking (R1) and physiological maturity (R6) in 2024 and 2025 under different nitrogen application regimes and plant densities. S, 75,000 plants ha^-^¹; D, 97,500 plants ha^-^¹. CK1 and SN1–SN4 denote treatments under S, whereas CK2 and DN1–DN4 denote treatments under D. CK, conventional single basal N application; N1–N4, split N application at different growth stages. Values are means ± SE (n = 3). Different lowercase letters indicate significant differences among treatments within the same year, density, and growth stage at P< 0.05.

Split nitrogen application increased nitrogen accumulation, with a stronger response at maturity than at silking (**Figure 5**). At silking, split-nitrogen treatments generally showed higher aboveground nitrogen accumulation than the corresponding controls, but treatment rankings varied between years and planting densities. At maturity, high-frequency split nitrogen application showed a clearer advantage, especially for total canopy and grain nitrogen accumulation. In 2024, SN4 increased total canopy and grain nitrogen accumulation by 83.8% and 68.6%, respectively, compared with CK1, while DN4 increased these two variables by 89.1% and 75.4%, respectively, compared with CK2. In 2025, SN4 and DN4 remained superior to their controls, with DN4 increasing total canopy and grain nitrogen accumulation by 90.4% and 86.6%, respectively, compared with CK2. These results indicate that, under the same total nitrogen input, the much higher canopy and grain nitrogen accumulation under SN4 and DN4 reflected improved synchronization between nitrogen supply and crop demand, as well as greater post-silking nitrogen acquisition and allocation.

### 3.6 Leaf senescence

#### 3.6.1 Relative green leaf area and its characteristic parameters

**Figure 6:**
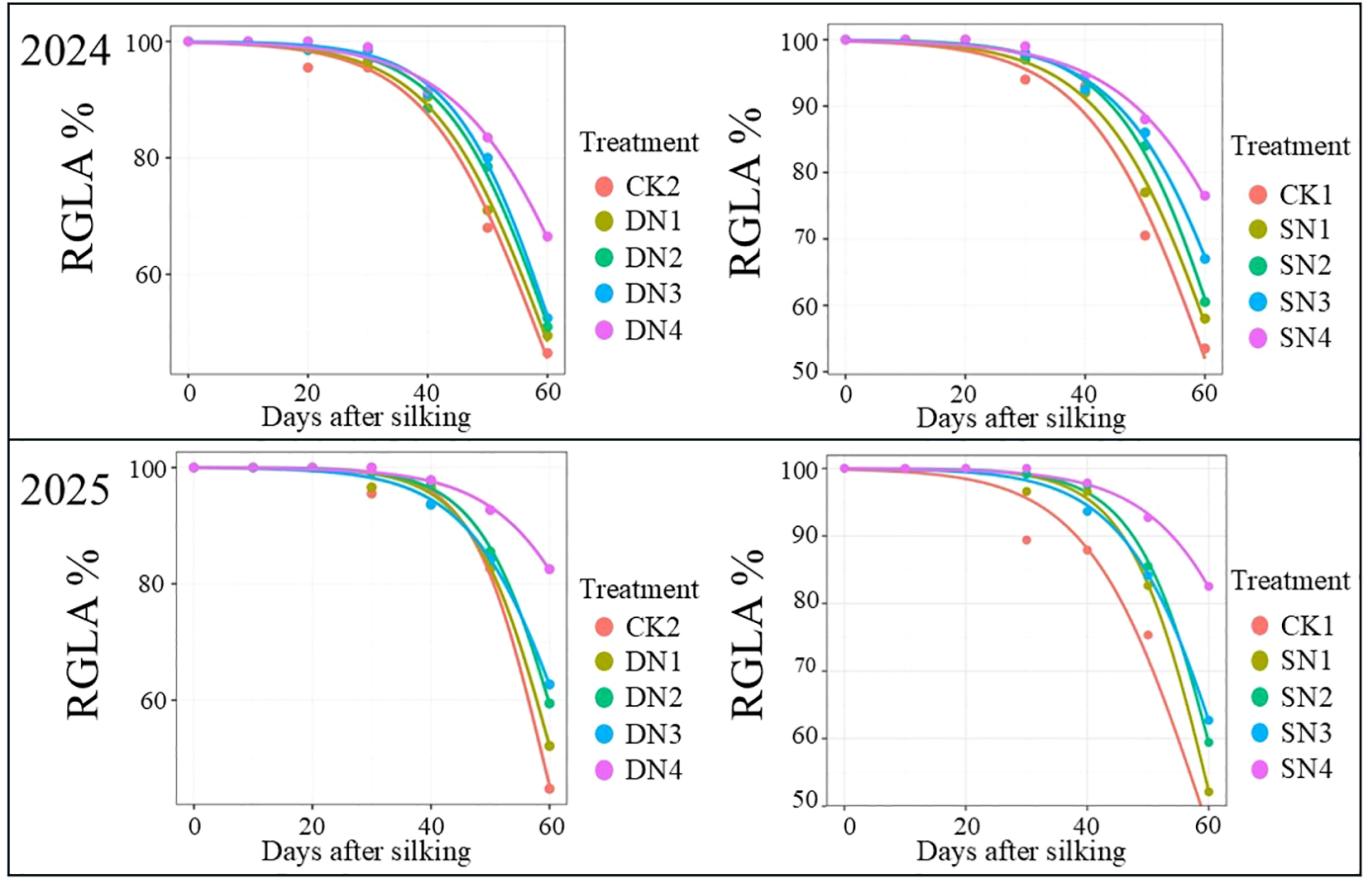
Logistic fitting curves of relative green leaf area after silking under different nitrogen application regimes and plant densities in 2024 and 2025. S, 75,000 plants ha^-^¹; D, 97,500 plants ha^-^¹. CK1 and SN1–SN4 denote treatments under S, whereas CK2 and DN1–DN4 denote treatments under D. CK, conventional single basal N application; N1–N4, split N application at different growth stages.

The coefficients of determination (R²) of the fitted Logistic equations were all greater than or equal to 0.90, indicating that the Logistic equation could adequately describe the dynamic process of post-silking leaf senescence under different treatments (**Figure 6**). The fitted Logistic parameters are presented in **Table 3**. With increasing nitrogen application frequency, the onset time of leaf senescence (Ts) and the time at which the maximum senescence rate occurred (Tmax) were delayed, whereas the mean senescence rate (V) and maximum senescence rate (Vmax) generally decreased. Based on the two-year averages, SN4 delayed Ts by about 2d compared with CK1 under density S, while DN4 delayed Ts by about 6d compared with CK2 under density D, suggesting a stronger numerical response of leaf senescence traits to the four-split treatment under the high-density condition. Meanwhile, compared with their corresponding controls, both SN4 and DN4 markedly delayed Tmax and reduced V and Vmax.

**Table 3:** Logistic parameters describing leaf senescence after silking under different nitrogen application regimes and plant densities in 2024 and 2025.

Year	Density (plants ha^-^¹)	Treatment	b	c	R^2^	T_s_ (d)	V(% d^-1^)	Vmax(% d^-1^)	Tmax (d)
2024	75,000	CK1	9.20	0.168	0.990	37.26	1.21	4.20	54.76
		SN1	9.10	0.17	0.988	36.24	1.23	4.25	53.53
		SN2	9.40	0.165	0.990	39.15	1.16	4.13	56.97
		SN3	9.15	0.162	0.991	38.33	1.17	4.05	56.48
		SN4	9.30	0.165	0.992	38.55	1.18	4.12	56.36
	97,500	CK2	9.30	0.175	0.985	36.34	1.25	4.38	53.14
		DN1	8.75	0.157	0.992	36.94	1.17	3.93	55.73
		DN2	9.00	0.16	0.990	37.88	1.16	4.00	56.25
		DN3	8.95	0.16	0.990	37.56	1.17	4.00	55.94
		DN4	9.10	0.145	0.993	42.48	1.05	3.63	62.76
2025	75,000	CK1	10.10	0.160	0.980	45.24	1.07	3.96	63.85
		SN1	8.18	0.120	0.980	45.00	0.90	2.91	70.31
		SN2	7.43	0.100	0.990	44.33	0.83	2.53	73.41
		SN3	8.64	0.110	0.990	50.33	0.85	2.83	76.33
		SN4	6.77	0.080	0.980	48.16	0.69	1.99	85.23
	97,500	CK2	9.84	0.170	0.990	41.25	1.15	4.18	58.88
		DN1	9.00	0.150	0.990	40.75	1.08	3.71	60.57
		DN2	9.12	0.150	0.990	42.37	1.05	3.64	62.57
		DN3	7.50	0.120	0.990	39.12	0.95	2.91	64.42
		DN4	8.04	0.110	0.990	47.05	0.85	2.71	74.24

S, 75,000 plants ha^-^¹; D, 97,500 plants ha^-^¹. Ts, onset time of leaf senescence; V, mean leaf senescence rate; Vmax, maximum leaf senescence rate; Tmax, time at which the maximum senescence rate occurred. R² represents the coefficient of determination of the logistic model.

#### 3.6.2 Antioxidant enzyme activities, MDA content, and soluble protein content in the ear leaf

**Figure 7:**
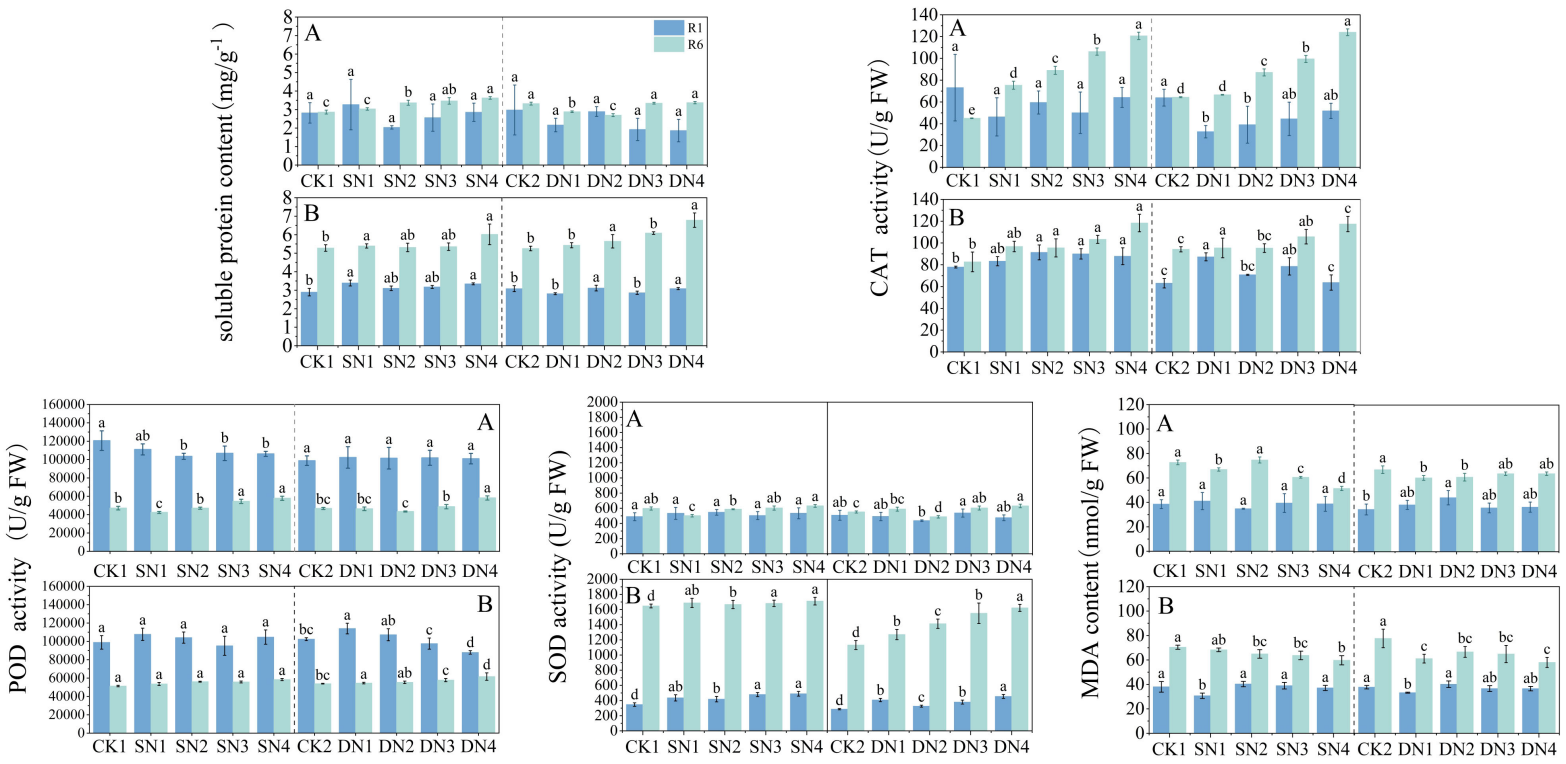
Effects of planting density and nitrogen application frequency on leaf physiological characteristics of maize at R1 and R6. Panels A and B represent 2024 and 2025, respectively. SP, soluble protein content; CAT, catalase activity; POD, peroxidase activity; SOD, superoxide dismutase activity; MDA, malondialdehyde content. R1, silking stage; R6, physiological maturity. CK1 and SN1–SN4 represent treatments under 75,000 plants ha^-^¹; CK2 and DN1–DN4 represent treatments under 97,500 plants ha^-^¹. Different lowercase letters indicate significant differences among treatments at the same growth stage within the same year at P< 0.05. Error bars represent standard error.

The effects of planting density and nitrogen application frequency on the physiological traits of the maize ear leaf showed similar stage-specific patterns, with stronger responses at maturity than at silking (**Figure 7**). MDA content was higher at maturity than at silking under both densities, indicating increased membrane lipid peroxidation during plant development. However, at maturity, MDA content generally decreased with increasing nitrogen application frequency, and SN4 and DN4 showed the lowest or relatively low levels, whereas changes at silking were smaller and less consistent. In contrast, antioxidant enzyme activities and soluble protein content generally increased with increasing nitrogen application frequency, especially at maturity. CAT activity was higher at maturity than at silking and increased with nitrogen application frequency, with N4 reaching the highest or near-highest level. POD activity was generally higher at silking than at maturity, but still increased with nitrogen application frequency at maturity. SOD activity and soluble protein content were generally higher at maturity than at silking, and both increased progressively with nitrogen application frequency, with the highest values under SN4 and DN4. Overall, increasing the frequency of nitrogen topdressing, especially to four applications, reduced MDA accumulation and enhanced antioxidant enzyme activities and soluble protein content in the ear leaf at maturity, thereby improving late-stage physiological activity and delaying leaf senescence.

### 3.7 Correlation analysis

**Figure 8:**
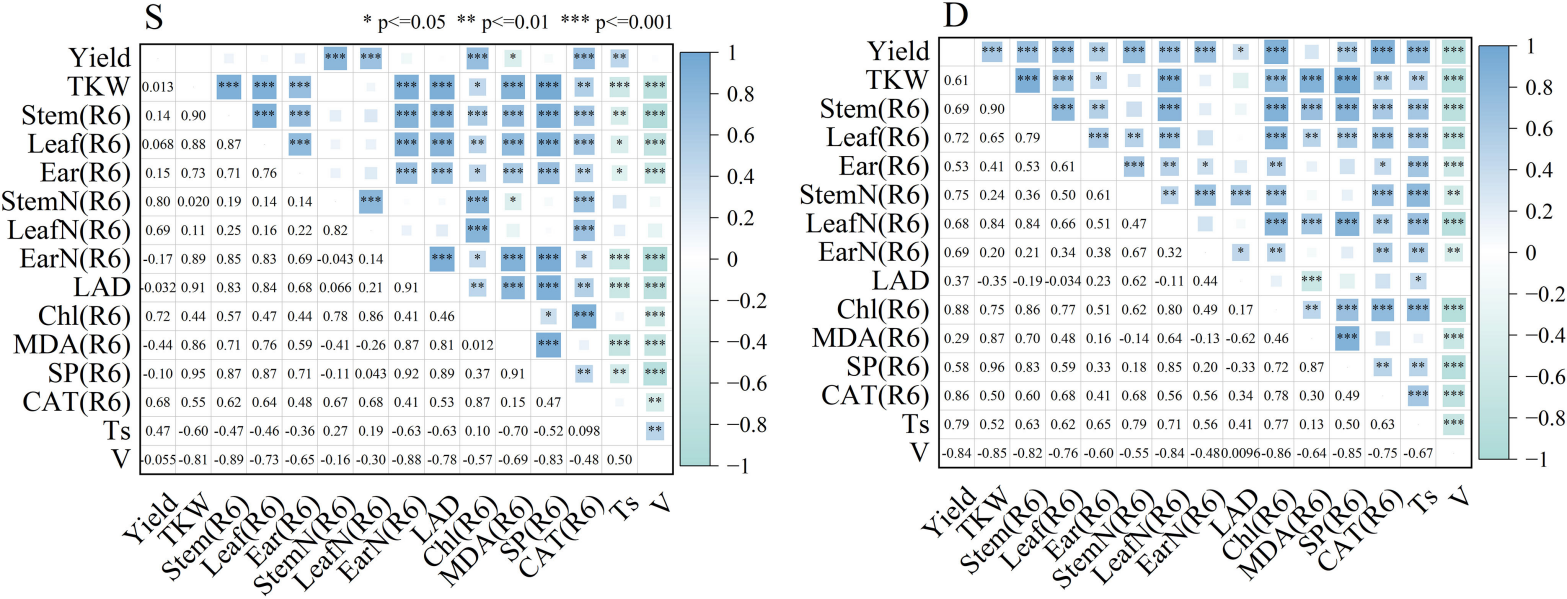
Pearson correlation analysis among yield, yield components, dry matter accumulation, nitrogen accumulation, leaf senescence traits, and physiological characteristics under different plant densities. S, 75,000 plants ha^-^¹; D, 97,500 plants ha^-^¹. Yield, grain yield; TKW, thousand-kernel weight; Stem(R6), Leaf(R6), and Ear(R6), dry matter weight of stem, leaf, and ear at physiological maturity, respectively; StemN(R6), LeafN(R6), and EarN(R6), nitrogen accumulation of stem, leaf, and ear at physiological maturity, respectively; LAD, leaf area duration; Chl(R6), chlorophyll content at physiological maturity; MDA(R6), malondialdehyde content at physiological maturity; SP(R6), soluble protein content at physiological maturity; CAT(R6), catalase activity at physiological maturity; Ts, onset time of leaf senescence; V, mean leaf senescence rate. Numbers indicate Pearson’s correlation coefficients. *, **, and *** indicate significance at P< 0.05, P< 0.01, and P< 0.001, respectively.

Under the 75,000 plants ha^-^¹ (S) density, grain yield was positively correlated with stem nitrogen accumulation, leaf nitrogen accumulation, chlorophyll content at maturity, CAT activity, and the onset time of leaf senescence (P< 0.01 or P< 0.001), whereas it was negatively correlated with MDA content (P< 0.05) (**Figure 8**). Under the 97,500 plants ha^-^¹ (D) density, grain yield was positively correlated with 1000-kernel weight, stem dry matter weight at maturity, leaf dry matter weight at maturity, ear dry matter weight at maturity, stem nitrogen accumulation, leaf nitrogen accumulation, ear nitrogen accumulation, post-silking photosynthetic potential, chlorophyll content at maturity, soluble protein content, CAT activity, and the onset time of leaf senescence (P< 0.05, P< 0.01, or P< 0.001), whereas it was negatively correlated with the mean senescence rate (P< 0.001).

## 4 Discussion

Increasing planting density is a major strategy for improving maize yield potential, but its yield effect depends on the balance between population-level ear number and single-ear productivity. In the present study, the higher planting density increased grain yield mainly by increasing the number of ears per unit area, whereas kernels per ear and 1000-kernel weight decreased. This indicates that dense planting expanded the population sink size but weakened the assimilate supply and grain-filling capacity of individual ears. Similar density-induced trade-offs between ear number and single-ear performance have been reported in previous studies (Olmedo Pico et al., 2023; Shao et al., 2024b; Wu et al., 2023; Zhao et al., 2025; Zhang et al., 2026a).

Split nitrogen application appeared to alleviate this source limitation during grain filling. Unlike planting density, split nitrogen application had limited effects on kernel number per ear, but it increased 1000-kernel weight and ear dry matter accumulation at maturity. This suggests that its yield advantage was mainly derived from improved post-silking source activity and grain filling rather than from an increase in sink number. Maize grain filling depends on current post-silking assimilate production and the remobilization of pre-silking stored materials (Barman et al., 2025; Liao et al., 2024). Therefore, the higher post-silking photosynthetic potential and greater ear dry matter accumulation under the four-split nitrogen treatment indicate that continuous nitrogen supply helped maintain source capacity and support sink filling under dense planting. This interpretation is consistent with previous reports that late or split nitrogen application can improve source–sink relations, optimize post-silking nitrogen accumulation, and promote grain filling and yield formation in maize (Deng et al., 2023; Wu et al., 2022; Xia et al., 2025; Zhang et al., 2026b).

Mechanistically, split nitrogen application may have better synchronized nitrogen supply with the reproductive-stage demand of densely planted maize. Compared with single basal nitrogen application, repeated nitrogen supply under drip fertigation likely prolonged root-zone nitrogen availability, supported post-silking nitrogen uptake, and improved nitrogen allocation during grain filling (Ciampitti and Vyn, 2011; Yan et al., 2017; Maltese et al., 2024). This continuous nitrogen supply may have maintained leaf nitrogen status and reduced premature nitrogen remobilization from functional leaves, thereby helping to sustain chlorophyll content, photosynthetic capacity, and delayed leaf senescence (Mu et al., 2018; Du et al., 2024b; Li et al., 2022a). At the same time, greater nitrogen accumulation in the ear may have provided nutritional support for kernel filling. Therefore, the four-split nitrogen treatment improved yield not simply by increasing total nitrogen accumulation, but by coordinating source maintenance, nitrogen remobilization, and sink filling under high-density conditions.

The stronger senescence-delaying effect of the four-split nitrogen treatment under high planting density may be explained by the greater physiological stress imposed by dense canopies. High planting density can accelerate canopy closure, reduce light availability in the middle and lower canopy, and intensify interplant competition for nitrogen during grain filling (Liu et al., 2023; Yan et al., 2017, 2024). These conditions may promote earlier nitrogen remobilization from functional leaves to developing grains, accelerate chlorophyll degradation, weaken antioxidant protection, and thereby advance leaf senescence (Mu et al., 2018; Lan et al., 2024; Du et al., 2024a; Li et al., 2022a). Under such conditions, the mismatch between a single basal nitrogen application and late-season nitrogen demand becomes more pronounced. In contrast, repeated nitrogen supply through drip fertigation may maintain root-zone nitrogen availability after silking, support post-silking nitrogen uptake, and help preserve leaf nitrogen status (Ciampitti and Vyn, 2011; Shen et al., 2025). This continuous nitrogen supply could sustain chlorophyll content and antioxidant enzyme activity, reduce membrane lipid peroxidation, and delay the decline in green leaf area. Therefore, the larger delay in Ts under DN4 than under SN4 may reflect that split nitrogen application more effectively alleviated the combined light and nitrogen competition caused by high planting density, thereby maintaining ear-leaf function during grain filling.

The physiological measurements in this study support this interpretation. Among the leaf senescence parameters calculated by the Logistic model, the onset time of leaf senescence (Ts) was significantly correlated with yield under both planting densities. In contrast, indicators such as the mean senescence rate showed a significant effect on yield only under the 97,500 plants ha^-^¹ (D) density, but not under the 75,000 plants ha^-^¹ (S) density. This indicates that, under split nitrogen application, yield variation under density D may be more closely related to the leaf senescence process (Yan et al., 2024; Li et al., 2025). However, the four-split treatment consistently delayed the onset of leaf senescence compared with the corresponding control under both densities. The ear leaf is a major photosynthetic source during the grain-filling stage and plays an important role in yield formation (Subedi and Ma, 2005). Under nitrogen deficiency during the late growth stage, maize leaves usually accelerate the breakdown of their own nitrogen-containing compounds and transport them to the grain, resulting in chloroplast disintegration and rapid degradation of chlorophyll and carotenoids (Wang et al., 2023a). In this study, split nitrogen application increased the photosynthetic pigment contents of the ear leaf at maturity, indicating that it helped maintain late-stage leaf photosynthetic function and supported its role in delaying leaf senescence. The results of this experiment also support this interpretation: the MDA content of the ear leaf at maturity gradually decreased with increasing split nitrogen application, and the four-split nitrogen treatment showed the lowest value, indicating that split nitrogen application substantially alleviated membrane lipid peroxidation damage. At the same time, CAT and SOD activities and soluble protein content in the ear leaf at maturity all increased with increasing nitrogen application frequency, and the four-split nitrogen treatment reached the highest or near-highest levels.

Although the four-split regime performed best under the conditions of this experiment, its practical adoption should also consider environmental and economic trade-offs. Under the same total nitrogen rate, split application may improve the synchronization between nitrogen supply and crop demand and potentially reduce nitrogen loss risk (Ren et al., 2022). Compared with conventional flood irrigation and manual fertilization, drip fertigation integrates water and fertilizer application, thereby reducing water use and labor demand. However, increasing fertigation frequency may still increase management and energy costs. Therefore, the large-scale adoption of the four-split regime should be evaluated together with nitrogen-use efficiency, environmental risk, and economic return.

In addition, this study was conducted using only the density-tolerant hybrid MY73. Because maize hybrids differ in stay-green ability, density tolerance, and nitrogen uptake and remobilization capacity, the magnitude of the response to split nitrogen application may vary among genotypes. Therefore, the four-split nitrogen regime identified here should be regarded as a reference for density-tolerant summer maize hybrids under drip fertigation, and its applicability to other genotype types requires further validation.

## 5 Conclusions

Split nitrogen application improved grain yield in densely planted summer maize. This advantage was associated with delayed post-silking leaf senescence, higher pigment content and antioxidant capacity in the ear leaf at maturity, lower membrane lipid peroxidation, and greater post-silking photosynthetic potential. Under high-density conditions, the senescence-delaying effect of split nitrogen application was more pronounced, and the yield response was numerically greater. Overall, under the conditions of this study, the combination of 97,500 plants ha^-1^ and four split nitrogen applications showed the best overall performance, and may provide a useful reference for high-yield cultivation of densely planted summer maize under drip fertigation in North China.

## Data Availability

The original contributions presented in the study are included in the article/supplementary material. Further inquiries can be directed to the corresponding author.
